# Supplementary material for: Peptide-Mediated Gene Transfer into Marine Purple Photosynthetic Bacteria
Source: Int J Mol Sci. 2020 Nov 16;21(22):8625. doi: 10.3390/ijms21228625 (PMC7697693; doi:10.3390/ijms21228625)
Supplement: Supplementary file 1 [file ijms-21-08625-s001.pdf]

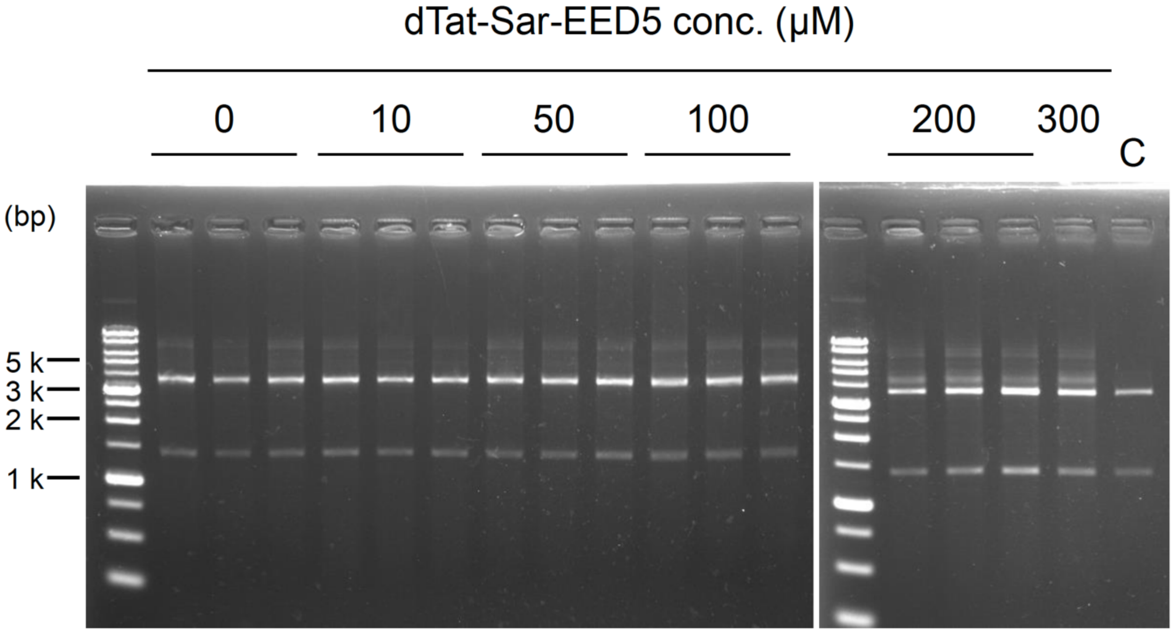

Figure S1. Plasmid extraction and restriction digestion of kanamycin resistant colonies of *R. sulfidophilum*. Plasmid solutions extracted from kanamycin resistant colonies were digested with *Bgl*II and *Hind*III and analyzed by 1% agarose gel. The pDNA delivery were carried out in the presence of different concentrations of dTat-Sar-EED5 (0, 10, 50, 100, 200, 300 µM).

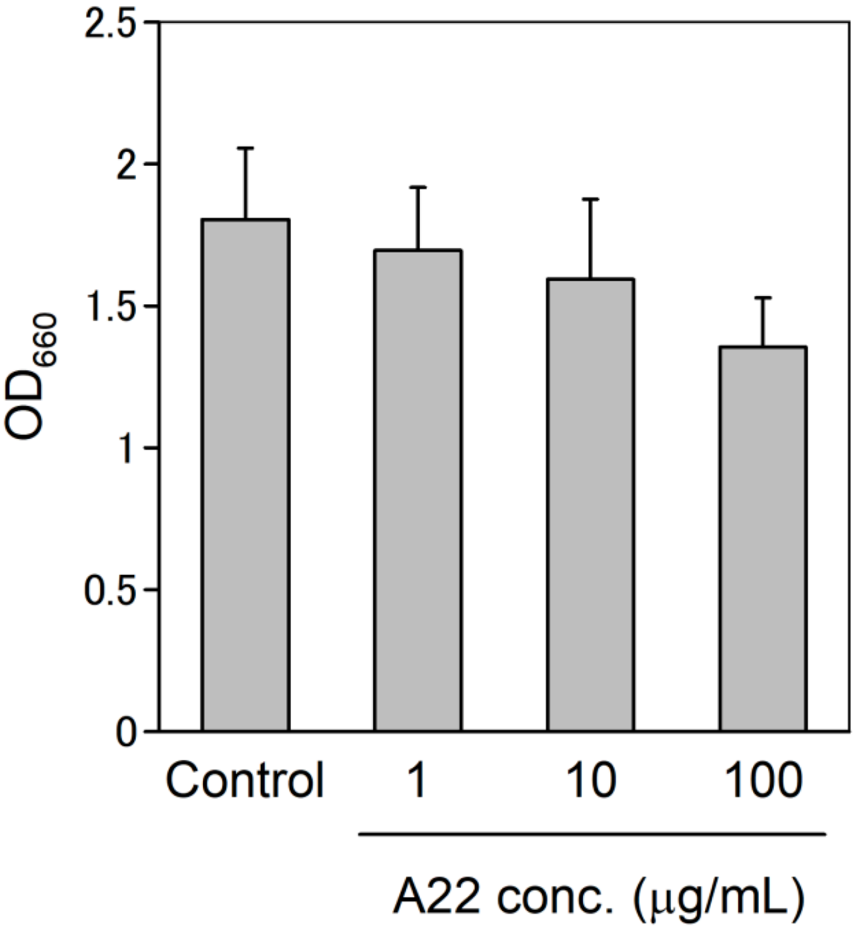

Figure S2. Effect of A22 on growth of *R. sulfidophilum*. *R. sulfidophilum* cells were incubated with different concentrations of A22 (1, 10 and 100 μg/mL) for 1.5 h. After removing the reaction mixture by centrifugation, cells were cultured in growth medium overnight and OD_{660} was measured. Data are the mean ± SD of at four cell cultures.

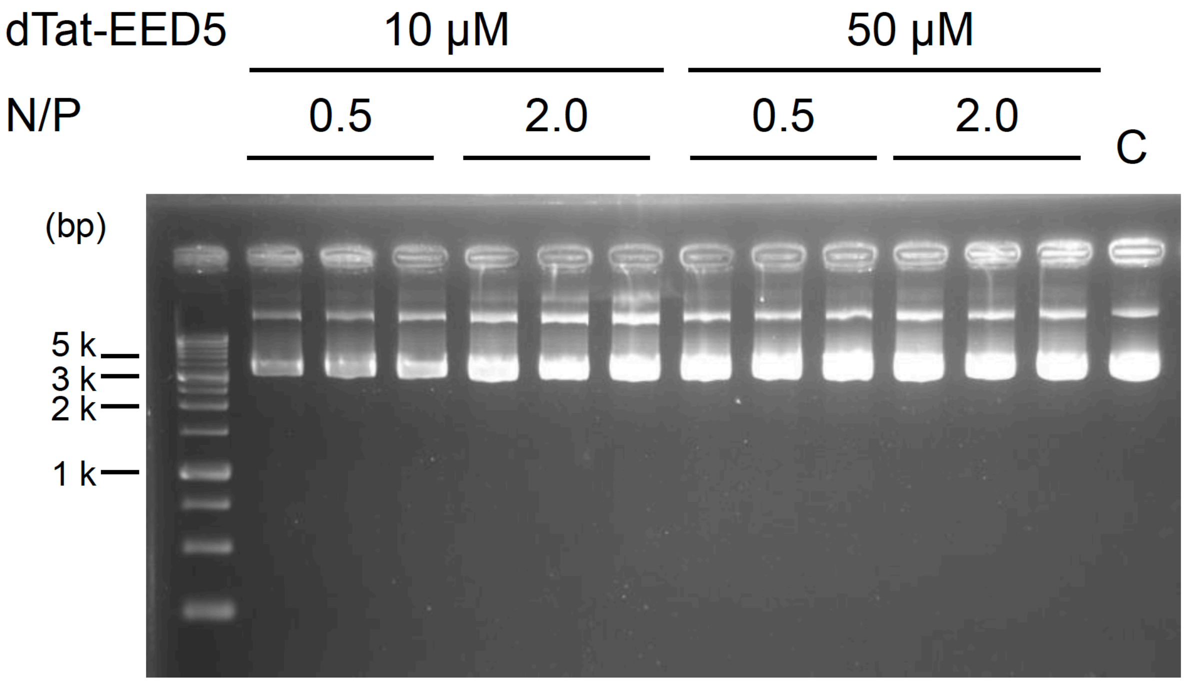

Figure S3. Plasmid extraction from ampicillin resistant colonies of *E. coli*. Plasmid solutions extracted from ampicillin resistant colonies were analyzed by 1% agarose gel. Experiments were carried out in the presence of 10 and 50 μM of dTat-Sar-EED5. N/P ratio was set to 0.5 and 2.0.
